# orthofisher: a broadly applicable tool for automated gene identification and retrieval

**DOI:** 10.1093/g3journal/jkab250

**Published:** 2021-07-15

**Authors:** Jacob L Steenwyk, Antonis Rokas

**Affiliations:** Department of Biological Sciences, Vanderbilt University , Nashville, TN 37235, USA

**Keywords:** phylogenomics, homology, orthology, Hidden Markov Model, gene family, sequence similarity search

## Abstract

Identification and retrieval of genes of interest from genomic data are an essential step for many bioinformatic applications. We present orthofisher, a command-line tool for automated identification and retrieval of genes with high sequence similarity to a query profile Hidden Markov Model sequence alignment across a set of proteomes. Performance assessment of orthofisher revealed high accuracy and precision during single-copy orthologous gene identification. orthofisher may be useful for assessing gene annotation quality, identifying single-copy orthologous genes for phylogenomic analyses, estimating gene copy number, and other evolutionary analyses that rely on identification and retrieval of homologous genes from genomic data. orthofisher comes complete with comprehensive documentation (https://jlsteenwyk.com/orthofisher/), is freely available under the MIT license, and is available for download from GitHub (https://github.com/JLSteenwyk/orthofisher), PyPi (https://pypi.org/project/orthofisher/), and the Anaconda Cloud (https://anaconda.org/jlsteenwyk/orthofisher).

## Introduction

Sequence similarity searches of genomic data are commonly employed in diverse fields of biology. Several pieces of software have been designed to infer statistically homologous sequences from databases of sequence data, such as BLAST, DIAMOND, and HMMER (Camacho *et al.* 2009; Eddy 2011; Madden 2013; Buchfink *et al.* 2015). One frequent use of sequence similarity search methods is for the identification of orthologs, sequences present in the common ancestor of two species, and homologs, sequences that stem from the same common ancestral sequence (Gabaldón and Koonin 2013). For example, the OrthoFinder software conducts BLAST all-*vs*-all searches across proteomes to infer groups of putatively orthologous genes (Emms and Kelly 2019). Similarly, the BUSCO software aims to identify putatively orthologous genes using a predetermined set of profile Hidden Markov Model sequence alignments (pHMMs) derived from single-copy orthologous proteins from the OrthoDB database (Waterhouse *et al.* 2013, 2018).

The results of these or similar pieces of software can facilitate diverse downstream analyses (Remm *et al.* 2001; Li *et al.* 2003; Train *et al.* 2017; Waterhouse *et al.* 2018; Emms and Kelly 2019). However, global analyses, such as those conducted by OrthoFinder, are computationally expensive and may be beyond the scope of a research project (*e.g.*, studies focused on a few genes). Similarly, software that rely on databases, such as BUSCO, are constrained to the orthologs therein. As a result, there is a need for bioinformatic software that can conduct automated identification and retrieval of putative homologs and orthologs across sequence databases using user-specified query sequences and output files that facilitate downstream analyses.

We introduce orthofisher, a command-line toolkit for automated identification of highly similar sequences across proteomes using custom pHMMs. orthofisher facilitates downstream analyses by creating multi-FASTA files populated with highly similar sequences identified during pHMM searches. Default parameters are designed to identify sequences with the highest sequence similarity (*i.e.*, putative orthologous genes), but users can customize its use to best fit their research question (*e.g.*, relaxed thresholds can be used to obtain all putatively homologous genes; similarly, searches in databases that contain gene isoforms can be used to retrieve all isoforms of a particular gene). We demonstrate the efficacy of orthofisher by evaluating the precision and recall for the identification of sequences with high similarity to query pHMMs in a multiple sequence FASTA (multi-FASTA) files from animals, plants, and fungi. Comparison of orthofisher, BUSCO, and OrthoFinder revealed similar performance in identification of sequences with high sequence similarity. Thus, orthofisher aims to streamline gene identification and retrieval from genomic data, which is the first step of many bioinformatic analyses and projects. We anticipate orthofisher will be of interest to diverse fields of computational biology and to biologists and bioinformaticians.

## Methods

**Figure 1:**
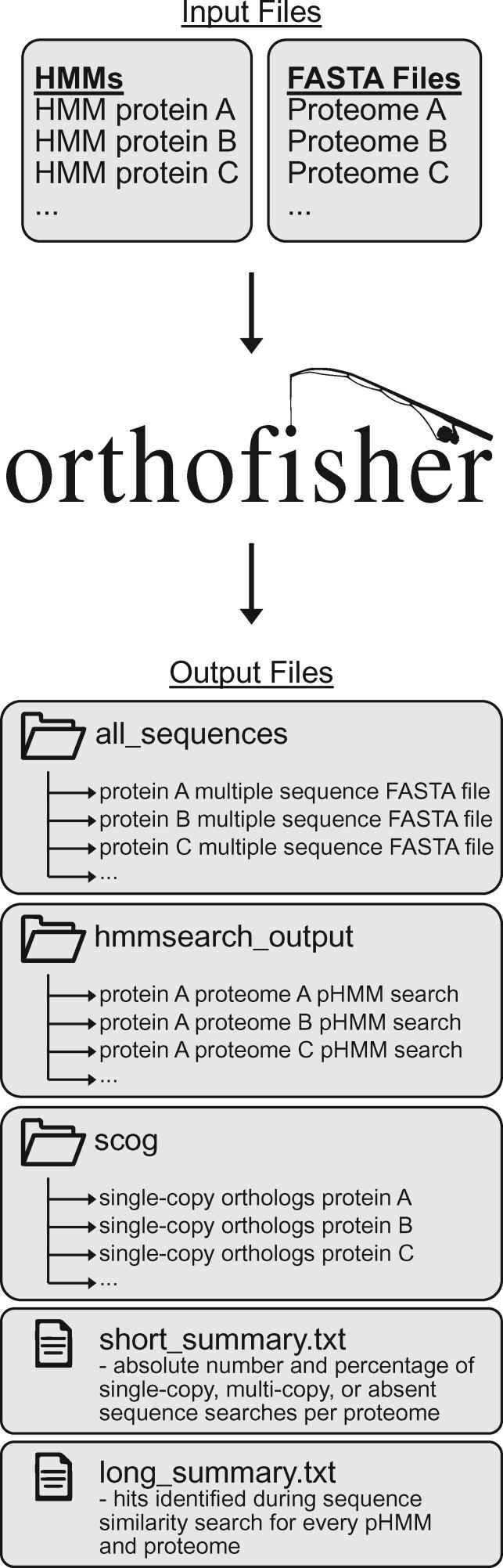
Workflow overview for orthofisher. orthofisher takes two files as input, which specify the location of query pHMMs and the FASTA files wherein sequence similarity searches will be performed. orthofisher then outputs three directories and two text files that summarize results and facilitate downstream analyses.

orthofisher requires two files as input (Figure 1). One file—specified with the -m, –hmm argument—provides the paths to query pHMMs that will be used during sequence similarity search; the other file—specified with the -f, –fasta argument—provides the paths to FASTA files that will be used as the sequence search database. orthofisher then loops through each FASTA file and uses each pHMM to search for similar sequences using HMMER3 (Eddy 2011) with an expectation-value threshold of 0.001 (which can be modified with the -e, –evalue argument). orthofisher then parses the resulting HMMER3 output using biopython (Cock *et al.* 2009) and identifies top hits. Top hits are defined following criteria used in the BUSCO pipeline (Waterhouse *et al.* 2018) wherein all sequences with scores that are greater than or equal to 85% of the score of the best hit are maintained. Users can modify this threshold using the -b, –bitscore argument. Top hits are considered homologous genes.

orthofisher outputs three directories and two text files that enable researchers to easily evaluate results from sequence similarity search and facilitate downstream analyses. The three directories are

- *hmmsearch_output*: HMMER3 output files,
- *all_sequences*: one multi-FASTA file per pHMM, which are populated with homologous sequences identified during the sequence similarity search step, and
- *scog*: one multi-FASTA file per pHMM, which are populated with only those homologous sequences that are present at most only once in each genome.

The two text files are

- *short_summary.txt*: the number and percentage of sequences present in single-copy, multicopy, or absent sequences per pHMM search and
- *long_summary.txt*: the homologous sequences identified during pHMM search for every query and sequence database.

Contents of output files will be heavily dependent on user parameters, the pHMMs used, and the input files. For example, transcriptomic data may require additional processing steps such as collapsing isoforms into a single-representative sequence per gene. The intent of orthofisher—which is to identify single-copy orthologous genes—is flexible enough to capture paralogous sequences as well. A tutorial for how to use orthofisher is publicly available as part of the online documentation https://jlsteenwyk.com/orthofisher/tutorial (last accessed: 2021-07-14).

Nearly 30% of bioinformatic tools fail to install (Mangul *et al.* 2019), which poses a nontrivial problem for the reproducibility of computational experiments. To remedy this issue, we implemented state-of-the-art standards of software development practices and design principles (Darriba *et al.* 2018) following previously established protocol (Steenwyk *et al.* 2020, 2021). For example, whenever changes to code are made, faithful function of orthofisher is tested using a continuous integration pipeline, a process that automatically builds, packages, and tests installation and function using Python versions 3.6, 3.7, and 3.8. We also wrote several unit and integration tests that span 95% of the orthofisher code.

## Results and discussion

To determine the similarities and differences between orthofisher and other algorithms that identify putative orthologs, we compared results obtained from orthofisher with that of BUSCO and OrthoFinder. BUSCO and OrthoFinder are both widely adopted methods of identifying orthologous genes across multiple proteomes. As noted in the introduction, each software differs—more specifically, BUSCO conducts homology searches using a predefined set of pHMMs and OrthoFinder conducts proteome-wide analysis to identify groups of orthologous genes. Thus, we expect that if orthofisher can identify putative orthologs across proteomes, it will identify the same genes BUSCO identifies during its sequence similarity search. Given that both algorithms conduct pHMM-based searches, we anticipate that both will exhibit near-identical performances. When comparing orthofisher and BUSCO to OrthoFinder, we anticipate the sequences identified during sequence similarity search by orthofisher and BUSCO will be in the same orthologous group of genes inferred by OrthoFinder.

### orthofisher and BUSCO obtain similar results

To evaluate the efficacy of orthofisher, we compared results obtained from orthofisher to those obtained from BUSCO, v4.0.4 (Waterhouse *et al.* 2018). To do so, both algorithms were used to identify 255 near-universally single-copy orthologous genes obtained from the Eukaryota OrthoDB, v10 (Waterhouse *et al.* 2013), database across the proteomes of animals (*Homo sapiens*: GCF_000001405.39; *Mus musculus*: GCF_000001635.27), plants (*Arabidopsis thaliana*, NCBI accession: GCA_000001735.2; *Solanum lycopersicum*: GCF_000188115.4), and fungi (*Saccharomyces cerevisiae*, NCBI accession: GCA_000146045.2; *Candida albicans*: GCA_000182965.3). Measures of precision and recall were calculated as follows:

$$\mathrm{Precision}=\frac{\mathrm{TP}}{\mathrm{TP}+\mathrm{FP}}$$

$$\mathrm{Recall}=\frac{\mathrm{TP}}{\mathrm{TP}+\mathrm{FN}}$$

where TP represents true positives, FP represents false positives, and FN represents false negatives of single-copy orthologous genes. Precision and recall values range from 0 to 1 and higher values reflect better performance.

Near perfect values of precision and recall {0.98 or [231/(231 + 4)] and 1.0 or [231/(231 + 0)], respectively} reveal orthofisher is able to automate the identification and retrieval of sequences with high similarity to the query pHMM. A low false-positive rate of 0.02 was observed. The difference in the performance of BUSCO and orthofisher stems from an additional set of gene-specific score and length thresholds used by the BUSCO software, which are not implemented in orthofisher. These results demonstrate that orthofisher can accurately identify homologous genes.

To demonstrate the importance of using a score threshold of 85% of the score observed in the best hit following the BUSCO pipeline (Waterhouse *et al.* 2018), we highlight an example where absence of a score threshold would have led to the identification of additional putatively orthologous genes. An HMMER search using the query BUSCO pHMM 1001705at2759 and an *e*-value threshold of 1e-10 in the proteome of *A. thaliana* reports the gene as multicopy whereas both orthofisher and BUSCO report this gene to be single copy. More specifically, when using only an *e*-value threshold of 1e-10, the following nine genes are reported: AEE76455.1, AEE78573.1, AEC10322.1, ANM68500.1, AED93406.1, AEE76521.1, AEE82221.1, AED98328.1, and AEE29324.1; however, AEE76455.1 has a score of 242.5 and the next best hit, AEE78573.1 has a score of 64.5. Thus, a score threshold of 85% of the best hit (in this case 242.5*0.85) is helpful during sequence similarity searches.

### orthofisher and BUSCO perform similarly to OrthoFinder

Comparison of the results of BUSCO and orthofisher to OrthoFinder, a global (or whole proteome) ortholog calling algorithm revealed BUSCO, orthofisher, and OrthoFinder produce similar results. To perform these comparisons, we first used OrthoFinder, v2.3.8 (Emms and Kelly 2019), to identify putative orthologous groups of genes in the same animal, plant, and fungal proteomes described above using an inflation parameter of 1.5 and DIAMOND, v0.9.24.125 (Buchfink *et al.* 2015). Then, we determined if genes identified as multicopy are part of the same or different orthologous group(s) of genes and also assessed if genes identified as single copy in BUSCO or orthofisher were also single copy in OrthoFinder.

Among multicopy genes, we found BUSCO and OrthoFinder had nearly identical performance in the proteomes of *A. thaliana*, *S. lycopersicum*, and *C. albicans*. For *S. cerevisiae*, one gene, 1545004at2759, out of 255 differed between BUSCO and OrthoFinder wherein BUSCO identified two homologs and OrthoFinder split these two genes into different orthologous groups of genes. A similar scenario was observed among 12/255 and 3/255 genes in the human and mouse proteomes, respectively. For orthofisher, a similar scenario was observed for 1/255 genes in *S. lycopersicum*; 1/255 genes in *A. thaliana*; 8/255 genes in *S. cerevisiae*; 4/255 genes in *C. albicans*; 13/255 genes in the human proteome; and 4/255 genes in the mouse proteome. We note that isoforms of the same gene sequence were present in the analyzed proteomes and were accounted for in these analyses.

Among single-copy genes, we observed a few instances where single-copy genes in BUSCO were multicopy in OrthoFinder. More specifically, this was observed for 8 genes in *S. lycopersicum*; 16 genes in *A. thaliana*; 2 genes in *S. cerevisiae*; 2 genes in *C. albicans*; 36 genes in the human proteome; and 26 genes in the mouse proteome. Similar results were observed for orthofisher. More specifically, 16/255 genes in *A. thaliana* were identified as single copy by orthofisher but were in multicopy orthologous groups of genes in OrthoFinder. The same observation was made for 7/255 genes in *S. lycopersicum*; 1/255 gene in *S. cerevisiae*; 2/255 genes in *C. albicans*; 35/255 genes in the human proteome; and 24/255 genes in the mouse proteome.

In summary, sequence similarity searches of 255 genes in 6 proteomes identified differences among 105 genes (6.86%; 105/1530) between BUSCO and OrthoFinder; similarly, we identified differences among 116 genes (7.58%; 116/1530) between orthofisher and OrthoFinder. These differences likely stem from differences in the approach of each algorithm to identify putative orthologs. Specifically, OrthoFinder uses DIAMOND and Markov clustering to identify orthologous groups, BUSCO uses pHMM-based search and gene-specific score and length thresholds using OrthoDB, and orthofisher uses pHMM-based similarity search thresholds. Also, these differences are in part driven by each algorithm reporting different results (*i.e.*, OrthoFinder reports groups of putatively orthologous genes and BUSCO and orthofisher report putative orthologous genes).

### orthofisher is helpful for estimating the number of members in a gene family

To demonstrate how to use orthofisher to estimate the number of gene family members, we estimate the number of DNA photolyase (PFam: PF00875) and zinc finger, C2H2 type (PFam: PF00096) homologs in *S. cerevisiae*, *C. albicans*, two species from the *Hanseniaspora* genus (*H. uvarum* NRRL Y1614 and *H. vineae* NRRL Y17529, both of which are known to lack DNA photolyases (Steenwyk *et al.* 2019)), and three *Aspergillus* species (*A. niger* CBS 513.88, *A. fumigatus* Af293, and *A. flavus* NRRL 3357). When estimating gene family number, we recommend lowering the score threshold to, for example, 25% of the best hit, which we have done here. In line with previous reports, we found that *Hanseniaspora* species lacked DNA photolyases whereas *S. cerevisiae*, *C. albicans*, and all *Aspergillus* species had one or two DNA photolyases. In contrast, proteins with Zinc finger domains are more abundant across all species with copies ranging from 16 (*H. vineae*) to 39 (*A. flavus*).

## Practical considerations

The intended use of orthofisher is to help identify orthologous genes across species using accurate and sensitive pHMM-based searches. We encourage users to evaluate results produced by orthofisher using additional approaches (*e.g.*, phylogenetic inference) to infer precise relationships of orthology and paralogy among sequences. We note that orthofisher is not explicitly designed to identify a single-representative sequence if multiple isoforms encoded by one gene sequence are present in a proteome. Thus, we also suggest users collapse isoforms prior to or after orthofisher analysis following standard protocol in many transcriptomics studies.

In summary, orthofisher is a command-line tool for automated identification and retrieval of genes of interest from genomic data. We anticipate orthofisher will be useful for evaluating genome completeness, performing phylogenomic inferences, estimating gene family size, and other analyses that rely on identification and retrieval of homologous genes from genomic data.

## Data availability

orthofisher comes complete with comprehensive documentation (https://jlsteenwyk.com/orthofisher/) (last accessed: 2021-07-14), is freely available under the MIT license, and is available for download from GitHub (https://github.com/JLSteenwyk/orthofisher) (last accessed: 2021-07-14), PyPi (https://pypi.org/project/orthofisher/) (last accessed: 2021-07-14), and the Anaconda Cloud (https://anaconda.org/jlsteenwyk/orthofisher) (last accessed: 2021-07-14). The proteomes, pHMMs, and outputs of orthofisher, BUSCO, and OrthoFinder are available through figshare (doi:10.6084/m9.figshare.14399150).
